# Targeting TGF-β Mediated SMAD Signaling for the Prevention of Fibrosis

**DOI:** 10.3389/fphar.2017.00461

**Published:** 2017-07-14

**Authors:** Kelly L. Walton, Katharine E. Johnson, Craig A. Harrison

**Affiliations:** Growth Factor Therapeutics Laboratory, Department of Physiology, Monash University, Clayton VIC, Australia

**Keywords:** fibrosis, TGF-β, activin, muscle, skeletal, myostatin, propeptide

## Abstract

Fibrosis occurs when there is an imbalance in extracellular matrix (ECM) deposition and degradation. Excessive ECM deposition results in scarring and thickening of the affected tissue, and interferes with tissue and organ homeostasis – mimicking an exaggerated “wound healing” response. Many transforming growth factor-β (TGF-β) ligands are potent drivers of ECM deposition, and additionally, have a natural affinity for the ECM, creating a concentrated pool of pro-fibrotic factors at the site of injury. Consequently, TGF-β ligands are upregulated in many human fibrotic conditions and, as such, are attractive targets for fibrosis therapy. Here, we will discuss the contribution of TGF-β proteins in the pathogenesis of fibrosis, and promising anti-fibrotic approaches that target TGF-β ligands.

## Introduction

In fibrotic disease, increased deposition of extracellular matrix (ECM) proteins compromises tissue architecture, and interferes with normal organ function. Fibrosis is frequently observed in the heart, liver, lungs, and kidneys, but can arise in any tissue that has suffered chronic insult. Triggers for fibrosis can be either biological (e.g., persistent bacterial/viral infections, genetic disorders or tissue injury) and/or environmental (e.g., pollutant/chemical exposure or allergens) (Macneal and Schwartz, 2012). Fibrosis is primarily driven by inflammatory cytokines including the interleukins (Nikolic-Paterson et al., 1996; O’Reilly et al., 2012), and members of the transforming growth factor-β (TGF-β) superfamily (Roberts et al., 1986; Meng et al., 2016), such as TGF-β1, activin A and activin B. Many of these ligands are expressed by infiltrating inflammatory cells, which migrate toward damage tissues. Of interest, these TGF-β ligands not only promote ECM deposition, but they also become concentrated within the accumulating matrix, thereby accelerating the pro-fibrotic response.

## TGF-β Ligands in Fibrosis

The TGF-β superfamily includes the TGF-β isoforms (TGF-β1, -β2 and -β3), activins and inhibins, growth differentiation factors (GDFs), bone morphogenetic proteins (BMPs), and anti-mullerian hormone (AMH). Although structurally similar, these ligands elicit distinct biological responses, based on their cell/tissue-specific expression, their interactions with inhibitory molecules and the unique combinations of receptors with which they complex. TGF-β ligands form receptor complexes with one of seven type I receptors (also termed activin-like kinase or ALK receptors) in combination with one of five type II receptors (ActRIIA, ActRIIB, TGFBRII, BMPRII, and AMHRII). For TGF-β1, ligand contact with the type II receptor TGFBRII leads to recruitment and phosphorylation of the type I receptor ALK-5. The BMPs, however, firstly contact their type I receptor (namely ALK3/ALK6), and then induce kinase activity toward the type II receptor BMPRII (reviewed in Miyazawa et al., 2002). In all instances, ligand-receptor complex formation leads to the activation of kinase domains within the receptors, which potentiate phosphorylation cascades involving SMAD transcription factors. The TGF-β isoforms and activins converge to induce intracellular signaling via SMAD-2/3 transcription factors. Activation of SMAD-2/3 regulates to the expression of several profibrotic genes, including collagens [*COL1A1, COL3A1, COL5A2, COL6A1, COL6A3, COL7A1*, (Verrecchia et al., 2001a,b)], plasminogen activator inhibitor-1 (*PAI-1*; Dennler et al., 1998; Hua et al., 1998), various proteoglycans (Schonherr et al., 1991; Romaris et al., 1995; Dadlani et al., 2008), integrins (Margadant and Sonnenberg, 2010), connective tissue growth factor (Chen et al., 2002), and matrix metalloproteases (MMPs; Yuan and Varga, 2001). BMPs via activation of SMAD-1/5/8, are capable of suppressing TGF-β mediated fibrotic gene expression (Wang and Hirschberg, 2003). As such, hyperactivation of activin/TGF-β-mediated SMAD-2/3 signaling promotes fibrosis, whereas increased BMP/SMAD-1/5/8 activity is likely anti-fibrotic. Here, we describe the contribution of TGF-β ligand induced SMAD signaling to the pathogenesis of human fibrosis, and emerging therapeutic strategies that target these ligands.

## Activators of the SMAD-2/3 Axis and Fibrosis

***TGF-**β** isoforms* –** TGF-β ligands that activate the SMAD-2/3 intracellular pathway have been heavily implicated in fibrosis. In particular, TGF-β1 is considered a major driver of human fibrotic pathologies. Circulating or tissue levels of TGF-β1 are elevated in human hepatic (Nagy et al., 1991), renal (Ketteler et al., 1994), and pulmonary fibrosis (Molina-Molina et al., 2006), as well as during cardiac failure (Khan et al., 2014). Exogenous TGF-β1 in rodents is sufficient to induce fibrosis in the lungs (Sime et al., 1997), and kidneys (Clouthier et al., 1997). Tissue specific pro-fibrotic activities of TGF-β1 are outlined in **Table 1**.

**Table 1:** Summary of evidence for TGF-β ligands in fibrosis.

LIGAND	Signaling pathway	ANTI/PRO-fibrotic	Liver fibrosis	Kidney fibrosis	Cardiac fibrosis	Muscle fibrosis	Lung fibrosis	Examples of therapeutic approaches
TGF-β1/ TGF-β2	TGF-β1 uses TGF-βRI (ALK5), TGF-βRII, and induces SMAD 2/3 signal. TGF-β2 also uses TGF-βRIII (betaglycan)	PRO	TGF-β1 induces α-SMA and type 1 collagen expression, and promotes migration/invasion of hepatic stellate cells (HSCs), the major producers of collagen in the liver (Presser et al., 2013). TGF-β2 accumulates in the bile ducts in human fibrotic liver disease, encouraging collagen deposition (Milani et al., 1991).	TGF-β1 drives differentiation of renal epithelial cells into α-SMA positive myofibroblasts, which also secrete collagen (Fan et al., 1999). In human glomerular disease, TGF-β1 protein expression is positively correlated with interstitial fibrosis severity and ECM production (Goumenos et al., 2001).	TGF-β1 and ECM production is upregulated following cardiac infarction in rats. Exogenous TGF-β1 can drive myocardial fibrosis *in vivo*. Cardiac fibroblasts differentiate into myofibroblasts in the presence of TGF-β1 (summarized in Lijnen et al., 2000).	High levels of TGF-β1 promote increased ECM deposition and attract inflammatory cells during muscle repair (summarized in Delaney et al., 2017). TGF-β1 expression is upregulated in dystrophic patients (Bernasconi et al., 1999).	TGF-β1 induces severe fibrosis in rat lungs, and is upregulated in patients suffering idiopathic pulmonary fibrosis (IPF) (Sime et al., 1997; Molina-Molina et al., 2006).	Small molecule inhibitor Pirfenidone^TM^ (Azuma, 2012) has been approved for the treatment of IPF. TGF-β1 targeting mAbs have also been tested in human IPF patients (Voelker et al., 2017), and TGF-β2 mAbs have anti-scaring activity in human glaucoma patients (Mead et al., 2003).

TGF-β3	TGF-βRI (ALK5), TGF-βRII, induces SMAD 2/3 signal	ANTI	TGF-β3 can alleviate the degree of hepatic fibrosis and tissue injury via the suppression of type 1 collagen synthesis (Zhang et al., 2010).			TGF-β3 is upregulated in muscle fibrosis, but competes with the pro-fibrotic TGF-β1 activity (Zhao et al., 2010).		Recombinant TGF-β3 (Juvista^TM^) demonstrated to improve wound healing during clinical trials (Ferguson et al., 2009).

Activin A	ActRIIA/ActRIIB, ALK4/7, induces SMAD 2/3 signal	PRO	Activin A is upregulated in rat models of liver fibrosis, and drives collagen production from hepatocytes (Sugiyama et al., 1998). Activin A is produced by activated HSCs *in vitro* and *in vivo*, and its activity appears to be unopposed by follistatin (Patella et al., 2006).	Activin A promotes cell proliferation, induces differentiation into myofibroblasts, and promotes expression of collagen in primary cultured renal interstitial fibroblasts in rats (Yamashita et al., 2004). Activin A expression is upregulated in multiple mouse models of chronic kidney disease (Agapova et al., 2016).	Heart failure patients have elevated activin A serum levels, which correlate with disease severity. Cardiomyocytes are the primary source of activin A production in the heart as it fails (Yndestad et al., 2004).	Activin A hyper-expression promotes muscle fibrosis, as evidenced by an increase in differentiated myofibroblasts and accompanied increase in ECM deposition (Chen et al., 2014).	Activin A expression is increased in cystic fibrosis (CF) patients, and activin inhibition reduces disease progression in a mouse model of CF (Hardy et al., 2015). Serum activin A and B concentrations are elevated in critically ill patients suffering acute respiratory failure (de Kretser et al., 2013).	Follistatin can improve muscle function and reduce fibrosis in two models of muscle disease (Mendell et al., 2015, 2017). Other ligands traps such as sActRII and propeptides work effectively in mouse models of activin-induced fibrosis (Chen et al., 2015; Agapova et al., 2016).
Myostatin	ActRIIA/ActRIIB, ALK4/5, induces SMAD 2/3 signal	PRO			Exogenous expression of myostatin from cardiomyocytes promotes interstitial fibrosis (Biesemann et al., 2015), and myostatin expression is increased following heart injury (Sharma et al., 1999). Aging hearts in myostatin null mice exhibit reduced fibrosis (Morissette et al., 2009).	Myostatin is a negative regulator of muscle mass and overexpression results in a pro-fibrotic response, with increased myofibroblasts and ECM production (Li et al., 2008).		Follistatin and soluble ActRII blocked myostatin signaling during clinical trials, leading to a reduction in fibrosis (Attie et al., 2013; Mendell et al., 2015; Campbell et al., 2016).

BMP7	BMPRII, BMPRI (ALK3/6), induces SMAD 1/5/8 signal	ANTI	BMP7 demonstrated to inhibit liver fibrosis and suppress activation of HSCs, via downregulation of TGF-β1 and α-SMA (Wang et al., 2014).	BMP7 expression decreases in renal fibrosis, and exogenous BMP-7 is protective in multiple animal models of nephropathies (summarized in Li et al., 2015).	BMP7 activates infiltrating monocytes into anti-inflammatory M2 macrophages, which inhibits apoptosis and fibrosis in prediabetic cardiomyopathy (Urbina and Singla, 2014).		BMP7 significantly reduced the progression of silica-induced fibrosis in rats, via upregulation of the SMAD1/5/8 axis and downregulation of SMAD2/3 signaling (Yang et al., 2013).	A BMP7 mimetic, AA123, demonstrated anti-fibrotic effects in a mouse model of kidney disease (Sugimoto et al., 2012).

BMP9	BMPRII, BMPRI (ALK1/5), induces SMAD 1/5/8 signal	PRO	BMP-9 promotes liver fibrosis via HSC differentiation, and promotes collagen 1 and fibronectin production (Bi and Ge, 2014; Breitkopf-Heinlein et al., 2017). BMP9 appears to mediate these actions through ALK1/5 (Munoz-Felix et al., 2016).					BMP-9 derivatives have been examined as bone regenerative agents (Bergeron et al., 2009, Bergeron et al., 2012). No specific BMP-9 intervention has been trialed to date for fibrosis therapy.

**FIGURE 1:**
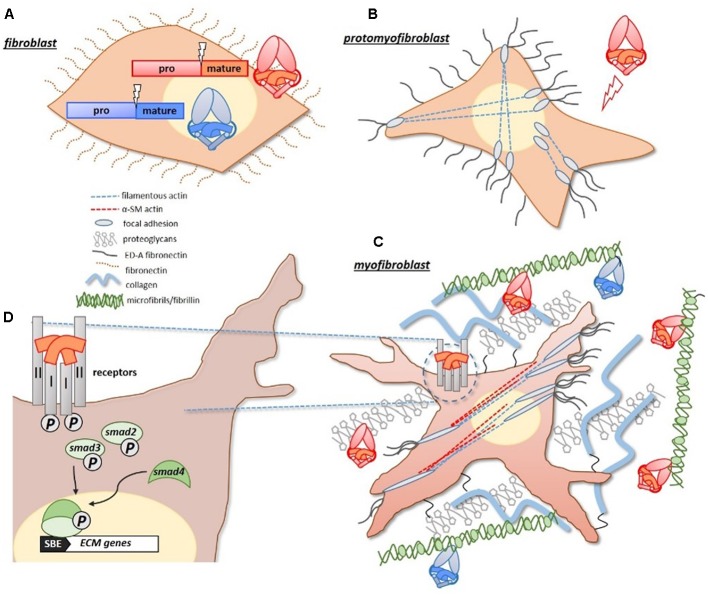
Pro-fibrotic activities of TGF-β proteins. **(A)** Pro-fibrotic TGF-β ligands, including TGF-β1 and activins (red/orange), and anti-fibrotic BMP ligands (blue) are similarly synthesized as large precursor proteins with pro-/mature-domains. **(B)** Under duress, quiescent fibroblasts differentiate into proto-myofibroblasts which express actin filaments that extend from a focal adhesion complex, and express extracellular ED-A fibronectin. **(C)** With increased exposure to TGF-β1, and ED-A fibronectin, proto-myofibroblasts become fully differentiated matrix-secreting myofibroblasts. The identifiable presence of α-smooth muscle actin (α-SMA), gives the myofibroblasts their measurable contractility. Both TGF-β1 and activins have a natural affinity for matrix proteins, such as fibrillin (microfibrils) and proteoglycans, and become concentrated in the expanding matrix of fibrotic tissue. The BMPs can also bind to fibrillin-containing microfibrils. **(D)** The activities of TGF-β1 and activins are initiated at the cell surface, where they form receptor complexes with type I/II receptors. Receptor activation leads to an intracellular phosphorylation cascade involving SMAD-2/3 transcription factors. SMAD-2/3 complex with SMAD-4, and translocate to the nucleus where they drive the expression of target matrix genes (via a Smad Binding Element, or SBE, in the promoter). Figure generated using Gabbiani (2003) as a guide.

Mechanistically, TGF-β1 promotes fibrosis by driving the differentiation of quiescent fibroblasts into matrix secreting myofibroblasts (Vaughan et al., 2000; Lijnen et al., 2003). Under duress fibroblasts differentiate into a proto-myofibroblast lineage, which in the presence of TGF-β1 (and ED-A fibronectin), become fully differentiated myofibroblasts (Gabbiani, 2003; **Figure 1**). Notably, TGF-β1 drives the production of α-smooth muscle (α-SM) actin, which gives the myofibroblasts their contractility. These cells represent activated fibroblasts, with a high synthetic capacity for ECM proteins (Ignotz and Massague, 1986). In response to heightened TGF-β1 signals, dominating myofibroblasts deposit excessive ECM, which compromises the local tissue architecture. TGF-β1 can further exacerbate this response by acting as a chemoattractant for some inflammatory cells (Wahl, 1992; Ludviksson and Gunnlaugsdottir, 2003), and is abundantly expressed by infiltrating macrophages in fibrotic tissue (Denholm and Rollins, 1993).

TGF-β1 is ubiquitously expressed within the body, and its target receptors (ALK5 and TGFβRII) have been identified on most cell types. As such, elevated levels of TGF-β1, either locally or systemically, can result in widespread manifestations of fibrosis. Acting through the SMAD-2/3 axis, TGF-β1 drives the expression of key ECM genes, including collagens (Verrecchia et al., 2001a), fibronectin (Hocevar et al., 1999), and PAI-1 (Dennler et al., 1998). These proteins are key components of developing fibrotic tissue. Additionally, TGF-β1 has a natural affinity for the ECM, binding directly to fibrillin microfibrils (Taipale et al., 1996). As such, TGF-β1 becomes concentrated in the accumulating ECM, thereby exacerbating the fibrotic response. TGF-β1 activity is kept in-check in healthy tissues by its naturally affiliated propeptide (termed ‘latency associated peptide’), which prevents TGF-β1 from complexing with its target receptors (Bottinger et al., 1996). For signaling to be initiated, an activation mechanism is required to break the propetide’s hold. For TGF-β1, activators include integrins (namely α_V_β_6_), thrombospondin-1 (TSP-1) and plasmin proteases (Lyons et al., 1990; Crawford et al., 1998; Munger et al., 1999; Morris et al., 2003). Significantly, in inflammatory conditions *de novo* expression of some/all of these activators increases (Zhang et al., 1999; Lopez-Dee et al., 2011), leading to activation and potentiation of TGF-β1 signaling (Popov et al., 2008). TGF-β1 activation is also increased in fibrotic tissue via biomechanical tissue stiffness, which causes force-dependant removal of the TGF-β1 propeptide (Wipff et al., 2007).

Although TGF-β1 is the best characterized pro-fibrotic factor within the family, TGF-β2 also displays potent fibrotic activity. TGF-β2 accumulates in the bile ducts in human fibrotic liver disease (Milani et al., 1991), and has been implicated in the fibrotic response associated with glaucoma (Wordinger et al., 2014). Remarkably, TGF-β3 appears to have anti-fibrotic activity in some tissues. TGF-β3 plays a key role in regulating epidermal and dermal cell motility during wound repair, a TGFβ-isoform-specific effect (Occleston et al., 2008). TGF-β3 is expressed at high concentrations during wound repair, and unlike TGF-β1 and -β2 isoforms, can promote wound healing without fibrotic scarring (Ferguson et al., 2009).

***Activins* –** Similar to TGF-β1, activins can trigger a pro-fibrotic response in several tissues via activation of the SMAD-2/3 cascade. Activins promote the proliferation of fibroblasts, their differentiation into myofibroblasts (Ohga et al., 1996; Ota et al., 2003; Yamashita et al., 2004), and the accumulation of ECM (Yamashita et al., 2004; Murakami et al., 2006). Serum concentrations of activin are elevated in patients suffering cystic fibrosis (Hardy et al., 2015), acute respiratory failure (de Kretser et al., 2013), chronic kidney disease (Agapova et al., 2016), and heart failure (Yndestad et al., 2004). Indeed, increased serum activin is a hallmark of many human chronic conditions and can have catastrophic consequences for affected patients. Our studies have shown that activins can drive the multi-organ wasting syndrome, cachexia (Chen et al., 2014), and that high levels of activin can induce a marked fibrotic response in skeletal muscle and liver, characterized by an influx of differentiated myofibroblasts and accompanied ECM deposition. Significantly, we have shown that the fibrotic pathology induced by activins can be fully reversed (Chen et al., 2014, 2015), highlighting the potential of anti-activin therapy to treat muscular dystrophies, in which patients suffer severe muscular fibrosis.

Activin is one of four major TGF-β ligands that signal through the activin type II receptors, ActRIIA/ActRIIB. Activin A, activin B, myostatin (GDF-8), and GDF-11 can all complex with ActRIIA/ActRIIB and initiate SMAD-2/3 intracellular activity. Despite this, all four ligands have non-overlapping bioactivities *in vivo* owing to their cell/tissue specific expression and distinct preferences for type I receptors (ALK4, ALK5 or ALK7). Myostatin is expressed almost exclusively in skeletal muscle, and can mimic the pro-fibrotic response observed under high activin conditions (Li et al., 2008). Myostatin promotes the proliferation of fibroblasts in muscle, and induces the expression of ECM proteins including collagen and fibronectin both *in vitro* and *in vivo* (Li et al., 2008). Given the structural homology of GDF-11 with myostatin (Walker et al., 2017) and shared receptor contacts, it is predicted that at high local concentrations GDF-11 will also exhibit pro-fibrotic activity.

Unlike the TGF-β isoforms, activins are secreted in an ‘active’ form. To constrain their activity, activins (and myostatin) are regulated extracellularly by follistatin. Follistatin binds directly to activin-related ligands, shielding their receptor contact sites so as to limit their signaling potential. Follistatin has opposing activity to activins/myostatin in fibrosis; attenuating early liver fibrosis (Patella et al., 2006), and lung fibrosis (Aoki et al., 2005) in murine models.

Like the TGF-β isoforms, activins have a natural affinity for the ECM – binding to heparin-sulphated proteoglycans (HSPGs) such as perlecan (Li S. et al., 2010). HSPGs are upregulated in many human fibrotic conditions, including human idiopathic lung fibrosis (Jiang et al., 2010; Westergren-Thorsson et al., 2017), Duchenne’s Muscular Dystrophy (Alvarez et al., 2002), liver disease (Roskams et al., 1996) and kidney fibrosis (Ebefors et al., 2011). Additionally, heparanase, the enzyme that metabolizes the carbohydrate chains on these proteoglycans, is also upregulated in fibrotic pathologies (Lv et al., 2016). Increased heparanase activity drives the release and activation of growth factors like activin and TGF-β (Masola et al., 2014). Consequently, activins affinity for HSPGs ensures an enriched pool of bioactive growth factors in the accumulating matrix.

## Activators of the SMAD-1/5 Axis and Fibrosis

Whilst hyper-activation of the SMAD-2/3 pathway is reported to be pro-fibrotic, signaling through the alternate SMAD-1/5 pathway is anti-fibrotic. Of all the SMAD-1/5 activators, BMP-7 has the most well documented anti-fibrotic activity. BMP-7 is expressed only in select adult tissues, including the kidney, and its expression declines in rodent models of renal fibrosis. BMP-7 counteracts TGF-β1 induced induction of myofibroblasts and ECM in multiple models of organ fibrosis (Zeisberg et al., 2003; Weiskirchen and Meurer, 2013). Addition of exogenous BMP-7 can both prevent and even reverse fibrosis in models of kidney disease (reviewed in Weiskirchen and Meurer, 2013; Li et al., 2015).

BMP-7 mediates its anti-fibrotic activity through the type I receptor, ALK-3 [also termed BMPRIA, (Sugimoto et al., 2012)]. Expression of ALK-3 increases in the early stages of kidney disease, and loss of ALK-3 exacerbates TGF-β1 mediated fibrosis, suggesting that ALK-3 is protective for fibrosis. ALK-3 is also a docking receptor for related BMP ligands, BMP-2, BMP-4 and BMP-6. Both BMP-2 and BMP-6 can similarly attenuate kidney fibrosis in rodent models of kidney disease (Yang et al., 2009; Dendooven et al., 2011), and BMP-6 expression is increased in humans and mice suffering non-alcoholic fatty liver disease (NAFLD) and is protective for hepatic fibrosis (Arndt et al., 2015). Interestingly, loss of BMP-4 is speculated to drive opposing pro-fibrotic response in cardiac tissue (Sun et al., 2013), and increased BMP-9 in the liver induces a fibrotic-like response (Breitkopf-Heinlein et al., 2017).

Like activins, BMPs are secreted from cells in an activated state. Once secreted, many of the BMPs are sequestered to the ECM by propeptide-mediated binding to fibrillins. Fibrillin binding creates a local concentration of BMPs and is thought to facilitate release of the active BMP proteins (reviewed in Wohl et al., 2016). Notably, perturbed fibrillin/microfibril assembly is a feature of human fibrosis (Wohl et al., 2016), and likely alters the bioavailability of associated BMPs. BMP activity is also restricted by extracellular antagonists Gremlin, DAN, Chordin and Noggin (reviewed in Weiskirchen et al., 2009).

## Targeting TGF-β Signaling for the Treatment of Fibrosis

TGF-β targeted therapies in fibrosis are designed to reduce activin/TGF-β signaling via SMAD-2/3, or alternatively, promote a BMP-mediated SMAD-1/5 signal. Many approaches are currently being explored, and some have reached the clinic (e.g., TGF-β3, follistatin, BMP-7). Leading approaches and emerging new strategies targeted to TGF-β mediated SMAD signals are described here.

***TGF-β targeted therapies* –** A plethora of TGF-β targeted therapies have been explored (reviewed in Akhurst and Hata, 2012), some of which have been designated specifically for fibrosis. These include, TGF-β1 antibodies for kidney fibrosis (Akhurst and Hata, 2012; Voelker et al., 2017), peptides (Llopiz et al., 2009), and receptor decoys for lung fibrosis (Yamada et al., 2007). Interventions for TGF-β2 mediated fibrosis include peptides for cardiac and skin fibrosis (Santiago et al., 2005; Hermida et al., 2009), and antibodies for glaucoma-related scarring (Mead et al., 2003). TGF-β3 in the form of Juvista^TM^ (Renova) was found to improve wound healing in phase I/II human clinical trials, but failed in phase III (Ferguson et al., 2009). However, few of these approaches have resulted in positive patient outcomes. Indeed, in a recent study testing a TGF-β1 specific antibody in a diabetic model of kidney fibrosis, disease progression was not improved (Voelker et al., 2017). Several factors likely impede the clinical effectiveness of these approaches, including the presence of multiple pro-fibrotic factors in advanced disease.

Despite their non-selectively, the small molecule inhibitors have advanced furthest owing to their economical production and ease of administration. Many of these approaches target the kinase activity of the TGF-β type I (TβRI, or ALK-5) and II (TGFβRII) receptors, including compounds GW788388 (Petersen et al., 2008) and SB-525334 (Grygielko et al., 2005), and have proven anti-fibrotic activities *in vivo*. Significantly, the small molecule inhibitor Pirfenidone^TM^ (Azuma, 2012) has been approved for the treatment of lung fibrosis in humans. Though encouraging, the promiscuous nature of these small molecule inhibitors renders them more prone to side effects (Noble et al., 2011).

Interventions that target the intracellular phosphorylation of SMAD-2/3 proteins, can also reduce TGF-β triggered fibrosis (reviewed in Munoz-Felix et al., 2015). SMAD-7, which sequesters SMAD-2/3, protects against renal fibrosis upon viral gene-delivery in a mouse model of kidney fibrosis (Terada et al., 2002). SIS3, a selective compound that targets only SMAD-3 proved to reduce the expression of ECM proteins and delayed the progression of diabetic nephropathy in a mouse model (Li J. et al., 2010).

***Activin and myostatin targeted therapies* –** Broad-spectrum TGF-β signaling inhibitors including SMAD-7, SIS3, and many of the small molecule kinase inhibitors can also block activin and myostatin induced signaling (Inman et al., 2002; Rodgers et al., 2014). The pleiotropic nature of these inhibitors is unfavorable for systemic use, and there is a pressing need for more tailored ligand therapies. ‘Ligand traps’ such as follistatin, soluble forms of the activin type II receptors (sActRII), and propeptides offer improved ligand selectivity, and their increased size favors serum retention (Wakefield et al., 1990).

Follistatin therapy is the leading approach for SMAD-2/3 blockade in activin/myostatin triggered fibrosis. Gene therapy approaches deploying follistatin have reached phase I/II clinical trials in humans (Mendell et al., 2015, 2017). Local expression of follistatin is sufficient to improve muscle function and reduce fibrosis in inflammatory myopathy (Mendell et al., 2017), and muscular dystrophy (Mendell et al., 2015). Similarly, the clinically relevant forms of sActRII (RAP-011 or ACE-011/sotatercept forms) can effectively reverse fibrosis in murine models of kidney disease (Agapova et al., 2016). However, clinical advancement of sActRII has been impeded by its apparent off-target vascular effects when used for human therapy (Attie et al., 2013; Campbell et al., 2016).

Propeptides, which are natural by-products of TGF-β assembly, and have an affinity for the ECM (Harrison et al., 2011), are attractive anti-fibrotic agents. We have shown that modified forms of the activin propeptides (propeptide-Fc fusion proteins) can both potently and specifically attenuate activin-mediated pathologies in mouse skeletal muscle (Chen et al., 2015, 2017). Similarly, we and others have found that the myostatin propeptide can revert muscle damage and attenuate fibrosis in mouse models (Qiao et al., 2008; Hamrick et al., 2010; Chen et al., 2017). This approach has also been demonstrated for the TGF-β propeptide, termed ‘latency associated propeptide’ or LAP, which has been shown to attenuate TGF-induced pathologies *in vivo* (Bottinger et al., 1996). However, as the TGF-β1 LAP has comparable affinity for all three TGF-β isoforms, and also GDF-8/-11, further modifications are required to improve its selectivity.

***BMP-targeted therapies for fibrosis* –** The anti-fibrotic activity of BMP-7 encouraged its application as a human therapy. However, like many TGF-β proteins, BMP-7 is poorly made and processed in mammalian cells (Swencki-Underwood et al., 2008), and upon delivery, is likely rapidly cleared from the blood (Coffey et al., 1987). To address these limitations, Sugimoto et al. (2012), developed a small molecule BMP-7 mimetic (AA123), which has the same anti-fibrotic activity as recombinant BMP-7 in a mouse model of kidney disease. A similar BMP-7 mimetic is currently in phase II clinical trials for acute kidney injury (Thrasos Therapeutics, Canada).

## Conclusion

Extracellular matrices provide a structural framework for cells and additionally serve as a scaffold for growth factors. Excessive ECM deposition, as observed in fibrosis, compromises tissue and organ structure and function and can lead to organ malfunction and failure. TGF-β proteins are major regulators of fibrosis, and the balanced activities of the pro-fibrotic and anti-fibrotic ligands ensure tissue homeostasis. TGF-β targeted therapies in fibrosis are designed to suppress the pro-fibrotic activity of TGF-β isoforms/activins/myostatin, or heighten the activity of anti-fibrotic BMPs. Promising anti-fibrotic TGF-β targeted therapies involve the use of ligand traps (follistatin, soluble receptors, propeptides) which sequester and deter activation of pro-fibrotic signals. Though encouraging, a major hurdle for clinical transition of these ligand traps is their *in vivo* stability, tissue-specificity, and minimisation of side effects. Indeed, one of the only drugs approved for TGF-β inhibition in lung fibrosis triggered gastrointestinal upset and the appearance of rashes (Noble et al., 2011) in treated patients, and activin blockade using the sActRII ligand trap caused vasculature complications (Attie et al., 2013; Campbell et al., 2016). Additionally, the importance of TGF-β ligands in non-fibrotic processes requires titrated treatments, so as not to ablate homeostatic functions. The ultimate goal for future TGF-β targeted fibrotic therapies is to identify ligand specific inhibitors with an extended signaling range that can act precisely within fibrotic tissue.

## Author Contributions

KW collected supporting evidence and wrote manuscript. KJ assisted with reference collection and collating. CH edited and provided guidance for the manuscript.

## Conflict of Interest Statement

The authors declare that the research was conducted in the absence of any commercial or financial relationships that could be construed as a potential conflict of interest. The reviewer MC and handling Editor declared their shared affiliation, and the handling Editor states that the process met the standards of a fair and objective review.
